# Evaluating the Effect of Resection on the Sealing Ability of MTA and CEM Cement

**Published:** 2012-08-01

**Authors:** Amin Salem Milani, Sahar Shakouie, Zahra Borna, Alireza Sighari Deljavan, Mohammad Asghari Jafarabadi, Fatemeh Pournaghi Azar

**Affiliations:** 1. Dental and Periodontal Disease Research Center, Tabriz University of Medical Sciences, Tabriz, Iran; 2. Department of Endodontics, Dental School, Tabriz University of Medical Sciences, Tabriz, Iran; 3. Department of Endodontics, Dental School, Urmia University of Medical Sciences, Urmia, Iran; 4. Dentist, Tabriz, Iran; 5. Tabriz Health Services Management Research Center, Dept. of Statistics and Epidemiology, Tabriz University of Medical Sciences, Tabriz, Iran; 6. Department of Operative Dentistry, Dental School, Tabriz University of Medical Sciences, Tabriz, Iran

**Keywords:** CEM Cement, Endodontic, Mineral Trioxide Aggregate, Retrograde Obturation

## Abstract

**Introduction:**

In cases of limited access to the surgical site, an alternative approach is to obturate the canal prior to surgery. Endodontic surgery is subsequently performed by root-end resection without retro-cavity preparation. This in vitro study was designed to compare the sealing ability of resected roots filled with either mineral trioxide aggregate (MTA) or calcium enriched mixture (CEM) cement.

**Materials and Methods:**

Seventy maxillary anterior teeth were selected. Following canal preparation, the teeth were randomly divided into four experimental (n=15) and two control (n=5) groups. In Group 1, CEM cement was placed into the apical 6-mm of the canal. The remainder of the canal was filled with gutta-percha/AH26 and 3-mm root-ends were resected. In Group 2: the teeth were treated as described above except that MTA was used instead of CEM cement. Group 3: The canals were obturated with gutta-percha/AH26. After root-end resection, retro cavities were prepared and filled with CEM cement. Group 4: The teeth were treated as described for group 3 except that MTA was used instead of CEM cement. The root apices of teeth were then placed in India ink, and maximum dye penetration was measured with a stereomicroscope. Nested ANOVA and Independent samples t-test were used to evaluate the statistical significance.

**Results:**

The mean dye leakage values for groups 1 to 4 were 402.6, 526.4, 141.0, and 177.4, respectively. The retrofilled groups had less microleakage compared to the resected materials; in the CEM cement groups this was statistically significant (P<0.05), i.e. root-end resection had no significant influence on the sealing ability of MTA, but significantly increased the microleakage of CEM cement (P=0.017). Overall, CEM cement showed less microleakage compared to MTA, however the difference was not significant.

**Conclusion:**

Within the limitations of this dye leakage study, we can conclude that if limited access prohibits retrofill placement, MTA or CEM cement can be used to fill the canal prior to root-end resection; as they have similar sealing ability. However, further laboratory and clinical studies are required to evaluate this alternative method.

## Introduction

Endodontic surgery is often indicated when non-surgical endodontic treatment is unsuccessful. It includes root-end resection, retro cavity preparation, and the placement of a root-end filling [1]. A variety of materials have been used as retrofills, including gutta-percha, zinc oxide-eugenol, amalgam, glass–ionomer cements, and other restorative materials [2]. Mineral trioxide aggregate (MTA) was first introduced as a root-end filling material. It has excellent biocompatibility and sealing ability [3] and is considered by many clinicians as the gold standard of endodontic material [4][5][6].

Recently, calcium enriched mixture (CEM) cement was introduced as a root-end filling material [7]. This cement consists mainly of CaO, SO_3_, P_2_O_5_ and SiO_2_. It releases calcium hydroxide during and after setting [7]. The antibacterial property of CEM is similar to that of calcium hydroxide and superior to MTA [8]. In comparison to MTA, this novel cement has superior properties such as increased flow, similar sealing ability, and decreased working time and film thickness [7][9]. CEM cement also has excellent biocompatibility [10][11] and low cytotoxicity, similar to MTA and significantly less than IRM [12][13]; showing favourable results in apexogenesis and pulpotomy of permanent teeth, management of furcal perforation, and internal and external root resorption [14][15][16][17].

The special consistency of MTA and CEM cement makes it difficult to deliver and compact into retro cavities, and the long setting time increases the possibility of washing out after surgery [18]. When there is limited access to the surgical site, an alternative approach includes obturation of the root canal prior to surgery. Following the setting of the materials, endodontic surgery is performed by resecting the root-end and exposing the set material without cavity preparation [19]. There are also some situations where MTA or CEM cement may be used to fill the entire root canal. In these instances, if endodontic surgery is subsequently required, the clinician may choose this alternative approach [19].

The primary concern regarding this approach is the sealing ability of resected MTA or CEM cement. An ex-vivo study showed that resection of set MTA has no effect on its sealing ability [19]. This was confirmed in a further investigation that showed root resection does not affect the sealing ability of MTA when a minimum 3 mm of MTA remains [20]. There is no published study evaluating the sealing ability of resected orthograde CEM cement. Therefore, this study was designed to compare the sealing abilities of resected roots filled with MTA or CEM cement.

## Materials and Methods

Seventy human maxillary anterior teeth with mature apices that were extracted due to periodontal disease were selected for this study. The inclusion criteria were a root length of at least 12 mm and an initial apical size no greater than ISO size 20. All teeth were cleaned free of attached tissues using periodontal curette, autoclaved and stored in 0.5% chloramine T solution until use.

### Sample preparation

The root length was standardised to 12 mm as measured from the apex using a diamond fissure bur mounted on a high speed handpiece (NSK, Japan). Coronal flaring was carried out using a RaCe rotary file size 40, 0.1 taper (FKG Dentaire, La-Cheaux-de Fonds, Switzerland). Hand stainless steel k-files (Maillefer, Ballagius, Switzerland) were used to enlarge the apical portion to ISO size 50. Ten millilitres of 5.25% sodium hypochlorite (NaOCl) was used to irrigate the canals during instrumentation. After instrumentation, 5 mL of 5.25% NaOCl was used to irrigate the canals followed by 5 mL of normal saline as final rinse. The canals were then dried using paper points. The prepared teeth were randomly divided into four experimental (n=15) and positive and negative control (n=5) groups. In Group 1, the root-ends of the teeth were placed on a moistened sponge to provide an apical stop. CEM cement (BioniqueDent, Tehran, Iran) was prepared according to manufacturer’s instructions, incrementally placed into the canal, and compacted using paper points and prefitted Schilder pluggers (Dentsply Caulk, Milford, DE, USA) until the apical 6 mm was filled. A cotton pellet moistened with phosphate buffered saline (PBS) was placed over the filling. The length and density of the filling were verified by radiography. After 24 hours incubation at 37˚C and 95% humidity, the remainder of the canal was filled with gutta-percha (GAPADENT Co., Ltd, Germany) and AH26 sealer (Dentsply; DeTrey, Konstanz, Germany) using a vertical compaction technique, and the apical 3 mm of the roots were resected perpendicular to the long axis of the teeth using a fissured diamond bur (Tizkavan, Tehran, Iran) mounted in a high-speed handpiece. Group 2: MTA (Angelus, Londrina, Brazil) (AMTA) was prepared according to the manufacturer’s instructions. The teeth were treated as described for group 1 except that AMTA was used instead of CEM cement. Group 3: The canals were obturated with gutta-percha and AH26 sealer using vertical compaction technique. After 24-hour incubation at 37˚C and 95% humidity, the apical 3 mm of the roots were resected. Subsequently, retrocavities were prepared to a depth of 3 mm using a Kis-3D microsurgical ultrasonic (Spartan, Missouri, USA) with medium power and water spray. The cavities were filled with CEM cement which was prepared according to the manufacturer’s instructions. The samples were then placed in a sponge moistened with PBS and incubated at 37˚C and 95% humidity for 24 hours. In Group 4, the teeth were treated as described for group 3 except that MTA was used instead of CEM cement.

In the positive control group, the canals were obturated with gutta-percha but without sealer. After 24-hour incubation, the apical 3 mm were resected, and retrocavities were prepared as described above. The canals in the negative control were obturated using gutta-percha/AH26. After 24-hour incubation, root-end resection and retrocavity preparation were performed. Finally, the cavities were filled with melted wax.

### Leakage test

All surfaces in the experimental and positive control groups, except the surface of the filling material, were covered by two layers of nail varnish. In the negative control, all root surfaces were covered by two layers of nail varnish. The root apices of all the teeth were then placed in India ink. After 72 hours, the teeth were rinsed and grooved on the buccal and lingual surfaces and split longitudinally into two sections. Maximum dye penetration was measured with a stereomicroscope (Carl Zeiss, Germany) at ×20 magnification to the nearest 0.1 µm.

### Statistical analysis

Statistical analysis was performed with SPSS windows version 16 (SPSS Inc, Chicago, IL). To evaluate statistical significance, nested ANOVA was used as the multivariate analysis and Independent samples were used as the t-test. The significance level was set at 0.05.

## Results

All the canals in the positive control group demonstrated leakage, conversely the canals in the negative control group did not display leakage. The results of the One-Sample Kolmogorov-Smirnov Test evaluating normality of data showed that data did not have normal distribution in the Resected orthograde MTA group (P<0.05). Hence, a logarithmic transform was applied to the data for subsequent analyses.

**Figure 1:**
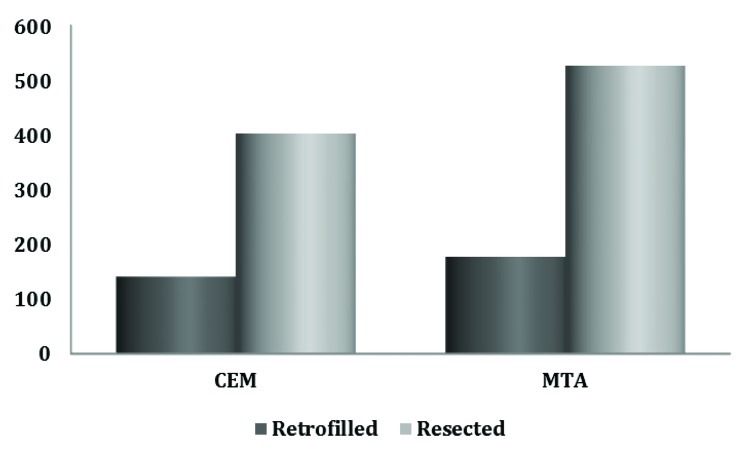
Bar chart showing mean apical dye leakage in MTA-CEM groups.

The resected orthograde materials showed more dye leakage than retrofilled materials, which was statistically significant in the case of CEM cement (P=0.017) (Table 1). CEM cement showed less microleakage compared with MTA in the resected or retrofilled state; however, the differences were not statistically significant (Figure 1).

**Table 1:** Summary of apical leakage (µm) in Resected and Retrofilled CEM and MTA

**Group**	**Mean (SD)**
**Resected CEM**	402.6 (293.4)
**Resected MTA**	526.4 (755.6)
**Retrofilled CEM**	141.0 (73.2)
**Retrofilled MTA**	177.4 (121.0)

## Discussion

There are circumstances where difficult access and isolation make placement of retrofilling materials very difficult. In these situations, a proposed alternative approach is to fill the canal coronally with retrofill materials prior to surgery. After setting, apicectomy is performed without any need for root-end preparation and retrofill placement [20].

Our study showed that resection does not significantly affect the sealing ability of MTA placed in an orthograde manner (P>0.05). This agrees with other studies [19][20], supporting the use of MTA. The present study revealed that resection increases the microleakage of CEM cement when this novel retrofilling material is used (Figure 1). However, another finding was that MTA and CEM have similar sealing abilities after resection (P>0.05). Therefore, CEM like MTA can also be used in this alternative approach.

In the present study, MTA and CEM were kept in contact with PBS, as a synthetic tissue fluid, during setting to simulate clinical situation [21]. Recent studies have shown that when MTA comes in contact with PBS, calcium ions released by MTA react with the phosphate in PBS leading to precipitation of carbonate apatite [22]. This is also true in the case of CEM cement; CEM has an endogenous source of both calcium and phosphate. Thus when MTA and CEM cement set in contact with non-phosphate fluid e.g. normal saline, only CEM cement is still able to produce apatite formation [21]. The high sealing ability of MTA and CEM has been thought to be due to precipitation of carbonate apatite when these materials come in contact with PBS in the MTA/CEM-dentin interfacial area and within dentinal [21][22]. This phenomenon may also be responsible for improving the sealing ability of MTA and CEM over time as shown in some recent studies [9][23][24][25][26][27]. Another explanation may be the slight expansion of MTA and CEM during setting [7][28][29]. In this study leakage was evaluated in the short term. If the present study analyzes long term results the differences in leakage of resected and retrofilled materials may be less than those observed in our study.

Resected Resilon, GP and MTA were compared in a similar study which showed significantly higher leakage values for resected Resilon and gutta-percha [30]. Since clinical studies are time consuming, expensive, and standardisation of the clinical parameters is difficult, using in vitro method to determine the sealing ability of filling materials seems logical [31]. Several in vitro methods have been used to evaluate the apical sealing ability of different root filling materials: dye penetration and extraction, fluid filtration, electrochemical, penetration of radioisotope tracers, and bacterial leakage methods [32]. Each technique has its advantage and disadvantages. Regardless of some of the drawbacks of dye leakage studies [33][34], this method is the most commonly used technique due to its simplicity, convenience and low cost [31]. CEM and MTA improve their seal over time, therefore the sealing ability of resected CEM or MTA should also be evaluated in the long term and with various leakage tests.

## Conclusion

Resected orthograde CEM cement and MTA have similar orthograde sealing abilities; therefore, if limited access and isolation impede retrofill placement both materials can be used to fill the canal prior to root-end resection.

## References

[1] Glickman GN, Hartwell GR, Ingle JI, Bakland LK, Baumgartner JC (2008). Endodontic surgery. Endodontics.

[2] De Bruyne MA, De Moor RJ (2009). Long-term sealing ability of Resilon apical root-end fillings. Int Endod J.

[3] Torabinejad M, Parirokh M. (2010). Mineral trioxide aggregate: a comprehensive literature review-part II: leakage and biocompatibility investigations. J Endod.

[4] Asrari M, Lobner D (2003). In vitro neurotoxic evaluation of root-end-filling materials. J Endod.

[5] Pistorius A, Willershausen B, Briseno Marroquin B (2003). Effect of apical root-end filling materials on gingival fibroblasts. Int Endod J.

[6] Sousa CJ, Loyola AM, Versiani MA, Biffi JC, Oliveira RP, Pascon EA (2004). A comparative histological evaluation of the biocompatibility of materials used in apical surgery. Int Endod J.

[7] Asgary S, Shahabi S, Jafarzadeh T, Amini S, Kheirieh S (2008). The properties of a new endodontic material. J Endod.

[8] Asgary S, Kamrani FA (2008). Antibacterial effects of five different root canal sealing materials. J Oral Sci.

[9] Asgary S, Eghbal MJ, Parirokh M (2008). Sealing ability of a novel endodontic cement as a root-end filling material. J Biomed Mater Res A.

[10] Asgary S, Eghbal MJ, Ehsani S (2010). Periradicular regeneration after endodontic surgery with calcium-enriched mixture cement in dogs. J Endod.

[11] Tabarsi B, Parirokh M, Eghbal MJ, Haghdoost AA, Torabzadeh H, Asgary S (2010). A comparative study of dental pulp response to several pulpotomy agents. Int Endod J.

[12] Mozayeni MA, Milani AS, Marvasti LA, Asgary S (2010.). Cytotoxicity of calcium enriched mixture cement compared with mineral trioxide aggregate and intermediate restorative material. Aust Endod J.

[13] Mozayeni MA, Milani AS, Marvasti LA (2009). Cytotoxicity of Cold Ceramic compared with MTA and IRM. Iran Endod J.

[14] Nosrat A, Asgary S (2010). Apexogenesis treatment with a new endodontic cement: a case report. J Endod.

[15] Nosrat A, Seifi A, Asgary S (2012). Pulpotomy in caries-exposed immature permanent molars using calcium-enriched mixture cement or mineral trioxide aggregate: a randomized clinical trial. Int J Paediatr Dent.

[16] Asgary S, Eghbal MJ, Ghoddusi J, Yazdani S (2012). One-year results of vital pulp therapy in permanent molars with irreversible pulpitis: an ongoing multicenter, randomized, non-inferiority clinical trial. Clin Oral Investig.

[17] Asgary S, Nosrat A, Seifi A (2011). Management of inflammatory external root resorption by using calcium-enriched mixture cement: a case report. J Endod.

[18] Kogan P, He J, Glickman GN, Watanabe I (2006). The effects of various additives on setting properties of MTA. J Endod.

[19] Andelin WE, Browning DF, Hsu GH, Roland DD, Torabinejad M (2002). Microleakage of resected MTA. J Endod.

[20] Lamb EL, Loushine RJ, Weller RN, Kimbrough WF, Pashley DH (2003). Effect of root resection on the apical sealing ability of mineral trioxide aggregate. Oral Surg Oral Med Oral Pathol Oral Radiol Endod.

[21] Asgary S, Eghbal MJ, Parirokh M, Ghoddusi J (2009). Effect of two storage solutions on surface topography of two root-end fillings. Aust Endod J.

[22] Reyes-Carmona JF, Felippe MS, Felippe WT (2010). The biomineralization ability of mineral trioxide aggregate and Portland cement on dentin enhances the push-out strength. J Endod.

[23] Martin RL, Monticelli F, Brackett WW, Loushine RJ, Rockman RA, Ferrari M, Pashley DH (2007). Sealing properties of mineral trioxide aggregate orthograde apical plugs and root fillings in an in vitro apexification model. J Endod.

[24] Bozeman TB, Lemon RR, Eleazer PD (2006). Elemental analysis of crystal precipitate from gray and white MTA. J Endod.

[25] Parirokh M, Askarifard S, Mansouri S, Haghdoost AA, Raoof M, Torabinejad M (2009). Effect of phosphate buffer saline on coronal leakage of mineral trioxide aggregate. J Oral Sci.

[26] Sarkar NK, Caicedo R, Ritwik P, Moiseyeva R, Kawashima I (2005). Physicochemical basis of the biologic properties of mineral trioxide aggregate. J Endod.

[27] Ghorbani Z, Kheirieh S, Shadman B, Eghbal MJ, Asgary S (2009). Microleakage of CEM cement in two different media. Iran Endod J.

[28] Chng HK, Islam I, Yap AU, Tong YW, Koh ET (2005). Properties of a new root-end filling material. J Endod.

[29] Storm B, Eichmiller FC, Tordik PA, Goodell GG (2008). Setting expansion of gray and white mineral trioxide aggregate and Portland cement. J Endod.

[30] Froughreyhani M, Milani AS, Rahimi S, Shakouie S, Fateh S (2011). Comparison of apical sealing ability of resected mineral trioxide aggregate, gutta-percha and a resin-based root canal filling material (resilon).. African Journal of Biotechnology.

[31] Sadighpour L, Rezaei S, Geramipanah F, Mohammadi M, Choubchian S (2010). Comparison of two techniques for evaluation of coronal leakage along of a glass fiber post. J Dent (Tehran).

[32] Verissimo DM, do Vale MS (2006). Methodologies for assessment of apical and coronal leakage of endodontic filling materials: a critical review. J Oral Sci.

[33] Wu MK, De Gee AJ, Wesselink PR (1994). Fluid transport and dye penetration along root canal fillings. Int Endod J.

[34] Ahlberg KM, Assavanop P, Tay WM (1995). A comparison of the apical dye penetration patterns shown by methylene blue and india ink in root-filled teeth. Int Endod J.

